# Inflammation in the Peripheral Nervous System after Injury

**DOI:** 10.3390/biomedicines12061256

**Published:** 2024-06-05

**Authors:** Dandan Gu, Yiming Xia, Zihan Ding, Jiaxi Qian, Xi Gu, Huiyuan Bai, Maorong Jiang, Dengbing Yao

**Affiliations:** 1 School of Life Sciences, Key Laboratory of Neuroregeneration of Jiangsu and Ministry of Education, Co-Innovation Center of Neuroregeneration, Nantong University, Nantong 226019, Chinahybai@ntu.edu.cn (H.B.); 2 Medical School, Nantong University, Nantong 226001, China

**Keywords:** inflammation, nerve regeneration, Wallerian degeneration, immune cells, cytokine, chemokine

## Abstract

Nerve injury is a common condition that occurs as a result of trauma, iatrogenic injury, or long-lasting stimulation. Unlike the central nervous system (CNS), the peripheral nervous system (PNS) has a strong capacity for self-repair and regeneration. Peripheral nerve injury results in the degeneration of distal axons and myelin sheaths. Macrophages and Schwann cells (SCs) can phagocytose damaged cells. Wallerian degeneration (WD) makes the whole axon structure degenerate, creating a favorable regenerative environment for new axons. After nerve injury, macrophages, neutrophils and other cells are mobilized and recruited to the injury site to phagocytose necrotic cells and myelin debris. Pro-inflammatory and anti-inflammatory factors involved in the inflammatory response provide a favorable microenvironment for peripheral nerve regeneration and regulate the effects of inflammation on the body through relevant signaling pathways. Previously, inflammation was thought to be detrimental to the body, but further research has shown that appropriate inflammation promotes nerve regeneration, axon regeneration, and myelin formation. On the contrary, excessive inflammation can cause nerve tissue damage and pathological changes, and even lead to neurological diseases. Therefore, after nerve injury, various cells in the body interact with cytokines and chemokines to promote peripheral nerve repair and regeneration by inhibiting the negative effects of inflammation and harnessing the positive effects of inflammation in specific ways and at specific times. Understanding the interaction between neuroinflammation and nerve regeneration provides several therapeutic ideas to improve the inflammatory microenvironment and promote nerve regeneration.

## 1. Background

In daily life, there is an extremely high incidence of nerve injuries due to various accidents such as car accidents, earthquakes, and surgeries, which subsequently trigger local pain, numbness, and in severe cases, even paralysis. These symptoms leave patients with a reduced quality of life and an increased economic burden [1]. According to epidemiology statistics, 551,612 patients presented with peripheral nerve injuries associated with exercise, sports, or recreation between 2009 and 2018 in the USA [2]. Therefore, increasing efforts have been invested in the field of repair and regeneration after nerve injury in order to alleviate the physical and psychological pain of patients and reduce this burden on society.

The nervous system can be divided into the central nervous system (CNS) and the peripheral nervous system (PNS). The main function of the PNS is to transmit nerve impulses to the CNS to form somatic or visceral sensations, and to transmit nerve impulses from the CNS to innervate somatic or visceral movements. After a peripheral nerve injury (PNI), the target tissue or structure that loses innervation not only loses its function, but also undergoes a series of changes such as atrophy and degeneration [1]. Unlike the CNS, the PNS has a strong ability to repair and regenerate itself [3]. PNI can cause the degeneration of distal axons and myelin sheaths, the phagocytosis of damaged cells by macrophages and Schwann cells (SCs), etc. Distal axons create a favorable regenerative condition for newborn axons after Wallerian degeneration (WD) [4].

The inflammatory response is a predominantly defense-based physiological response of the body to harmful stimuli, a protective behavior in which multiple cells are involved. Traditionally, neuroinflammation refers to any inflammatory response that occurs in the CNS, especially in the brain and spinal cord. However, it has also been suggested that neuroinflammation can expand to the PNS, especially the sciatic nerve and dorsal root ganglia (DRG) [5]. Immune cells, cytokines, chemokines, and second messengers are the main mediators of the inflammatory response. Immediately after an injury, the infiltration of immune cells and the activation of resident cells begin. In the PNS, the pro-inflammatory period is short, followed by an anti-inflammatory period of injury healing [6]. The range of inflammatory responses depends on a variety of factors. The age of the host, the site of injury, the duration, and the intensity of the stimulus are the main factors influencing the body’s inflammatory response [7]. Therefore, neuronal responses to axonal injury are largely dependent on the environment. The injury site, severity, and host’s age are just a few of the factors that influence the ability of axonal regeneration. The activation of intrinsic neuronal mechanisms, as well as a supportive extracellular environment, can significantly promote repair and regeneration after a nerve injury [8].

Neuroinflammation is a condition in which the levels of pro-inflammatory and anti-inflammatory factors in the body increase dramatically after an injury, while activating neuroglial cells such as microglia and astrocytes to cause an immune response, thereby resulting in symptoms such as inflammation and pain [9]. Neuroinflammation can be categorized into acute and chronic phases. In the acute phase, inflammation helps in nerve repair and the phagocytosis of unwanted cells, thus achieving protection, while in the chronic phase, neuroinflammation is highly likely to lead to the deterioration of injured neurons [8]. Neuroinflammation is a self-protective mechanism of an organism, through which the nervous system protects itself from pathologic injury [8]. It has been shown that the mechanisms of neuroinflammation contribute to nerve repair [10], the most prominent of which is the phagocytosis of tissue debris by immune-associated cells and the massive secretion of nerve growth factor to create a favorable microenvironment for neuronal axon regeneration after nerve injury. For example, the WD after a PNI is a specific series of cellular and molecular events distal to the injured nerve fibers and is thought to be an innate immune response or neuroinflammation [11]. The cellular and molecular events progress toward denervated target tissues in nerve fibers that are remote from the lesion site. Cellular events include the degeneration of the distal end of severed axons, the activation of SCs, the disruption of the blood–nerve barrier, and the recruitment of blood-derived macrophages and other types of cytokine- and chemokine-producing immune cells [12]. In addition, axonal injuries trigger molecular changes primarily related to inflammation, including the regulation of the extracellular matrix (ECM) within the nerve by metalloproteinases, the upregulation of the neurotrophic factor, and cytokine production [13,14]. Both cellular and molecular events in the distal end of injured nerves create conditions for inducing axonal regeneration in the proximal stump.

However, the role of inflammation in the PNS is more controversial. On the one hand, inflammatory responses in damaged nerves and their mediators play an important role in the process of nerve regeneration. On the other hand, these also develop conditions for the induction of neuropathic pain (NPP). Therefore, a detailed understanding of the inflammation after a traumatic nerve injury and during nerve regeneration is important for improving functional recovery after peripheral nerve reconnection/reconstruction and for tissue engineering [8]. In addition, the early suppression of the inflammatory response after nerve injury to minimize NPP may lead to the deceleration or cessation of axonal regeneration.

## 2. Inflammatory Response after Nerve Injury

### 2.1. Wallerian Degeneration

**Figure 1:**
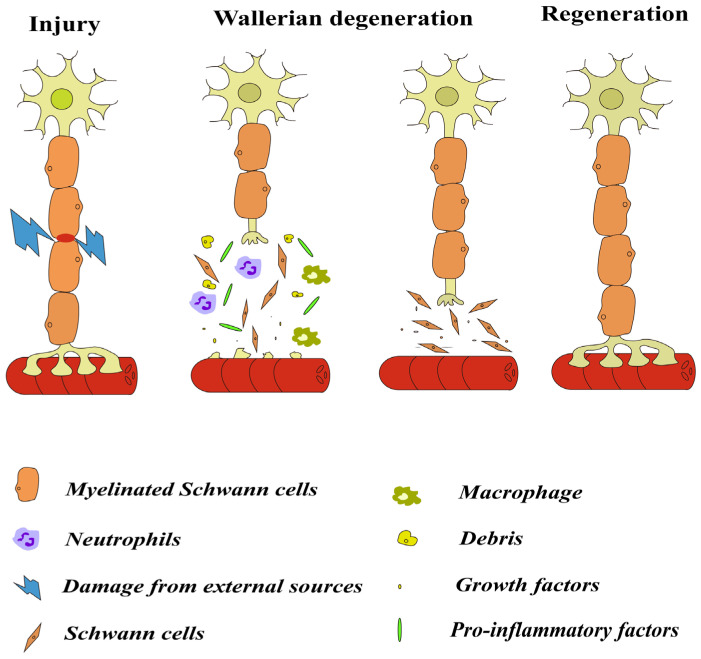
Wallerian degeneration (WD) and axon regeneration after peripheral nerve injury (PNI). After PNI, the distal nerve fibers are detached from the cell body and begin to degenerate. Then, Schwann cells (SCs) proliferate, migrate, produce cytokines/trophic factors, and phagocytose debris. The resident macrophages are activated and recruited macrophages and neutrophils clear myelin and debris. In the end, the axon is able to traverse the injury site and connect with targets, and myelinating SCs remyelinate the regenerated axon.

After nerve injury, the distal nerve fibers at the damaged site are detached from the cell body that is the center of nutrition and metabolism, and the distal nerve fibers, including the nerve endings, begin to degenerate. This process is known as WD, which includes a series of changes such as the denaturation and disintegration of axons and myelin sheaths, the proliferation of SCs, the infiltration of macrophages and mast cells, and the removal of axon and myelin fragments (Figure 1) [15,16]. Nerve injury usually triggers an inflammatory response in which the innate and adaptive immune systems are activated by endogenous signals that originate from damaged or necrotic cells distal to the damaged nerve [11]. The inflammatory response following nerve injury is indispensable for nerve repair and functional reinnervation [17].

WD is a relatively rapid and complex process in the PNS, triggering structural changes in distal myelin sheaths and axons within a few hours of injury, followed by gradual disintegration, increased phagocytosis, and the removal of damaged myelin and axons from the lesion over the next few days [18]. WD is also a process in which multiple active substances and cells are involved and axonal disintegration actively occurs [19,20]. During the phase of WD, SCs in vivo are activated to dedifferentiate, while immune-related cells such as neutrophils and macrophages are recruited to the lesion site [21]. The removal of tissue debris provides the necessary space for nerve regeneration and also reduces the inhibitory factors for myelination production. Dedifferentiated SCs proliferate in large numbers, form Büngner’s bands in the basal lamina canal, secrete a variety of neurotrophic factors that promote neuronal survival, and provide the nutrients necessary for nerve regeneration, as well as promoting axonal growth. Therefore, WD is a prerequisite for determining axonal regeneration and functional recovery after nerve injury [22].

### 2.2. Cells Involved in Inflammation after Injury

The inflammatory response activates macrophages, neutrophils, lymphocytes, and makes these cells accumulate at the wound site, thereby causing neuronal death, glial scarring, axonal degeneration, etc. [23]. After injury, neutrophils and macrophages intervene in the intracellular endogenous antioxidant system, leading to cell death [24]. For a severe injury, overcoming the local hypoxia and tissue necrosis due to inflammation is the prerequisite for nerve repair and regeneration, so a favorable microenvironment is necessary to facilitate angiogenesis as well as the proliferation and migration of neuroglial cells (e.g., SCs) in the absence of necrotic tissue debris [25].

#### 2.2.1. Macrophages

Macrophages are widely found in the lungs, liver, and nervous system, and play a key role in maintaining homeostasis, repairing wounds, and tissue regeneration [26]. In the PNS, macrophages can be divided into resident macrophages and infiltrating macrophages. Resident macrophages exist stably in the nervous system, while infiltrating macrophages are attracted by chemokines to the injured area and distal nerve segments after PNI [27]. After PNI, the distal stumps of axons in the injured segment need to be removed to facilitate axonal regeneration, and macrophages participate in axonal regeneration through the phagocytosis of the axon fragments, the disintegration of myelin sheaths, and the secretion of cytokines and growth factors [6].

Macrophages rapidly respond to nerve injury by activating and accumulating at the site of the nerve injury, exerting potent phagocytosis and secretory functions to create a favorable local microenvironment for nerve regeneration [28]. Studies have confirmed that during WD, infiltrating macrophages are mainly responsible for myelin degradation and debris removal, and resident macrophages are mainly involved in axonal regeneration [29]. In the early stage of nerve injury, M1 macrophages mainly dominate the pro-inflammatory response; in the middle and late stages of nerve injury, they are gradually replaced by M2 macrophages that suppress the inflammatory response and promote nerve regeneration through the secretion of cytokines [28].

Macrophage polarization plays an important role in the repair of peripheral nerve injuries [30]. M1 macrophages are involved in the development of neuroinflammation and pain after PNI through the high expression of proinflammatory cytokines, such as tumor necrosis factor-α (TNF-α), interleukin (IL)-6, and IL-1β. M2 macrophages are usually considered to be beneficial to nerve repair after PNI. The M2 polarization-driven process is closely related to the regression of inflammation. M2 macrophages attenuate inflammation by highly expressing anti-inflammatory cytokines such as IL-10 and transforming growth factor-β (TGF-β). After PNI, M1 macrophages polarize to become M2 macrophages, which can secrete more TGF-β with neuroprotective effects and inhibit the neuroinflammatory microenvironment, thus promoting nerve regeneration.

After PNI, the local hypoxia and tissue necrosis secondary to inflammation impede nerve repair and regeneration [27]. Macrophages respond to hypoxia by inducing angiogenesis to provide nutrients and promote SC migration and proliferation to remove necrotic tissue. This ability of macrophages is important for PNS injury repair and regeneration. Angiogenesis provides nutrient support for nerve repair and contributes to the recovery of long-distance peripheral nerve defects. Vascular endothelial growth factor (VEGF-A) is an essential angiogenic factor for peripheral axon growth, and macrophages are an important source of VEGF-A. Macrophage-derived VEGF-A can initiate angiogenesis, increase vascular permeability, and promote endothelial cell proliferation and migration to enhance revascularization, which is essential for axonal regeneration and functional recovery after nerve injury [31].

SCs are key cells in WD. The growth arrest specific 6 (GAS6) secreted by macrophages and polarization to the M2 phenotype can promote migration, the proliferation of SCs, maturation and myelin regeneration, as well as axonal extension [32]. After nerve injury, macrophages usually infiltrate the injured area earlier than SCs to construct regenerative channels, and they control the ECM in neural bridging by upregulating the axon guidance molecule Plexin-B2 to provide an appropriate substrate for the directed migration of SCs in neural stumps [33].

Every coin has two sides. Macrophage migration activity is not always favorable for nerve injury repair. The migration of excess infiltrating macrophages has the potential to cause more proinflammatory factors to be released, thereby stimulating the local microenvironment and causing further tissue destruction. Macrophages may also exacerbate NPP by releasing pro-inflammatory mediators that trigger pain [34]. The reduced migration and infiltration of macrophages into the DRG lead to a decrease in the level of the pro-inflammatory factor IL-6 in the local area, thereby alleviating neuromechanical pain. This suggests that macrophage migration promotes NPP after PNI [35].

#### 2.2.2. Neutrophils

Neutrophils, as myeloid leukocytes, are the first line of defense of the intrinsic immune system and account for 50–70% of all circulating leukocytes in the body [36]. The body has a large reserve capacity for neutrophil production, which can rapidly double in number during situations such as acute infections. As an important component of intrinsic immunity, neutrophils have a variety of immune defense functions in the traditional sense. Currently, phagocytosis, degranulation, and the generation of neutrophil extracellular traps (NETs), which has received close attention in recent years, have been studied [37].

A well-known function of neutrophils is their phagocytosis; however, this phagocytosis is often overlooked in the field of neurobiology [38]. This is because after nerve injury, it is often assumed that macrophages in the PNS and microglia in the CNS exert a phagocytic effect on myelin and cellular debris. And, the phagocytic effect of neutrophils is similar to that of macrophages/microglia [38]. Neutrophils have been shown to be necessary for the removal of myelin sheaths in the distal sciatic nerve after sciatic nerve transection injury. C-C Motif Chemokine Receptor 2 (CCR2) knockout mice without infiltrating macrophages displayed a similar rate of myelin clearance to that of wild-type (WT) mice 7 days after sciatic nerve transection [39]. Further studies by Lindborg using CCR2 knockout mice demonstrated that after sciatic nerve injury, neutrophils accumulated at the site of injury and exhibited phagocytosis; in WT mice, the number of neutrophils was greatly reduced and myelin debris clearance was significantly reduced after the use of an anti-Ly6G antibody [40].

Researchers have identified the immunomodulatory role of neutrophils that release cytokines and chemokines to initiate adaptive immune responses. These factors are released into the extracellular space or through exosomes. Neutrophils produce IL-1β and TNF-α, which promotes the production of cytokines by other cells to direct immune cells to the site of injury. Upon entering the site of injury, neutrophils recruit monocytes by expressing C-C Motif Chemokine Ligand 2 (CCL2), CCL3, CCL19, and CCL20 [41]. The reduced cytokine release from neutrophils can decrease immune cell activation, thereby altering the immune response in neurological disease.

Neutrophils also secrete neutrophil peptide-1 (NP-1), a member of the defensin polypeptide. NP-1 recruits monocytes and macrophages [42], and it has been demonstrated that NP-1 promotes functional recovery following sciatic nerve injury [43]. Nerve injury causes changes in gut flora, and a recent study has shown that changes in gut bacterial metabolites significantly contribute to changes in neutrophil infiltration, thereby influencing peripheral nerve regeneration [44].

#### 2.2.3. Lymphocytes

T lymphocytes are the last immune cells that reach the site of the injured nerve. In a rat model of chronic constriction injury, T lymphocytes infiltrated the injured sciatic nerve 3 days after injury and then peaked 14–28 days after injury [45]. T lymphocytes contribute to the immune response in the late stage of injury by producing proinflammatory or anti-inflammatory cytokines that support both cellular and humoral immunity. Type 1 helper T (Th1) cells secrete proinflammatory cytokines (e.g., TNF-a, interferon-γ) that activate adjacent macrophages, neutrophils, and natural killer (NK) cells, whereas anti-inflammatory cytokines (e.g., IL-4, IL-10) released by Th2 cells inhibit a variety of macrophage functions and inhibit/regulate proinflammatory cascade responses [46]. After a facial neuron crush injury, less facial motor neurons survived in both STAT-6 knockout mice (an impaired Th2 response) and RAG-2 knockout mice (a lack of mature T and B cells) compared with WT mice; T-Bet knockout mice (impaired Th1 response) exhibited delayed neurologic recovery [47]. These findings indicate that Th1 and Th2 cells are necessary for axonal regeneration in peripheral nerves.

#### 2.2.4. Schwann Cells

SCs are the most abundant neuroglia in the PNS and are classified as either myelinated and unmyelinated. SCs constitute the blood–nerve interface (or the blood–nerve barrier) that provides metabolic support to axons and regulate the response to nerve injury [48]. SCs with a high differentiation plastic potential can redifferentiate and dedifferentiate in response to injury and disease, and actively participate in regenerative and inflammatory processes. Thus, SCs, under physiological and pathological conditions, are critical for axonal function [49].

Nerve injury triggers the transformation of myelinated and unmyelinated SCs into a cellular phenotype that specifically promotes nerve repair, which provides an environment for axonal regeneration after injury [50]. The injury-induced reprogramming of SCs involves the downregulation of myelinating genes and the activation of a series of axon repair-related genes including the upregulation of trophic factors, the elevation of cytokines as part of the intrinsic immune response, myelin sheath clearance through the activation of myelin autophagy in SCs and macrophage recruitment, and the formation of regenerative tracks that guide axons to their target sites [51].

To investigate the molecular mechanism of SCs interacting with the inflammatory response after nerve injury, Amanda et al. [52] used immunopanning techniques to obtain SCs 3, 5, and 7 days after sciatic nerve injury and performed RNA-sequencing. The expression of genes related to cytoskeletal rearrangement peaked on the 5th day after injury [52].

SCs and fibroblasts in peripheral nerves express major histocompatibility complex type I (MHC-I) molecules, which play an important role in triggering immune rejection after non-autologous nerve transplantation [53]. These cell surface molecules provide intracellular proteins to cytotoxic CD8^+^ T cells, specifically activating and damaging recognized foreign cells [54]. The time course of MHC-I expression in neurons is associated with axonal regeneration after PNI, which demonstrates that this cell surface molecule plays an important role in nerve regeneration [55]. MHC-II delivers antigens from genetically distinct individuals to helper CD4^+^ T cells, triggering the late rejection of non-autologous tissue transplants. MHC-II is expressed on antigen-presenting cells (APCs), such as macrophages. Although SCs express low amounts of MHC-I but not MHC-II, significant elevations of MHC-II molecules are detected on their surface stimulated by the synergistic action of interferon-γ with TNF-α and in the presence of activated T cells [56]. Thus, SCs have the ability to process and deliver antigens in order to become facultative APCs under certain specific conditions.

The functions of the above-described cells are summarized in Table 1.

### 2.3. Factors Involved in Inflammation

#### 2.3.1. Cytokines and Chemokines

Cytokines are low-molecular-weight soluble proteins secreted by lymphocytes, macrophages, etc. Cytokines regulate immune cell homeostasis in inflammation by mediating innate and adaptive immunity, and serve as biomarkers for many diseases [57,58]. Cytokines are classified as either pro-inflammatory or anti-inflammatory factors according to their role, with IL-1β and TNF-α being typical pro-inflammatory factors and IL-10 and others being typical anti-inflammatory factors [59].

IL-1 is a potent activator of its own expression and release as well as a major mediator in inflammation that regulates the inflammatory response [60,61,62]. IL-1β is a typical proinflammatory factor that is directly involved in the inflammatory response and promotes the activation of nuclear factor-κB to induce the expression of NLRP3 proteins and inflammatory factors, including IL-1β, ultimately leading to a sustained inflammatory response [63]. IL-1β is first transcribed as an inactive precursor (pro-IL-1β) and concomitantly initiates inflammasomes. Pro-IL-1β is processed by NLRP3 inflammasome and caspase-1, leading to the extracellular formation of IL-1β [64]. Studies have shown that IL-1β is closely associated with many inflammation-related diseases, such as neurodegenerative diseases, rheumatoid arthritis (RA), and chronic obstructive pulmonary disease (COPD) [65,66].

TNF-α is a key component of immunity and shows strong pro-inflammatory activity to promote the secretion of several pro-inflammatory mediators [67]. TNF-α activates the immune system and promotes nerve regeneration after nerve injury. In TNF-α receptor knockout mice, nerve recovery after PNI was slower [68]. However, the inappropriate or excessive production of TNF-α can be harmful and may lead to diseases. For example, TNF-α is upregulated in patients with Parkinson’s disease (PD), which may serve as a biomarker for PD.

IL-10 is an immunomodulatory cytokine that effectively inhibits the secretion of pro-inflammatory factors (e.g., TNF-α and IL-1β) and the expression of MHC, and suppresses the activity of immune cells. IL-10 is produced by macrophages and B-cells and binds to the IL-10 receptor (IL-10R) on macrophages and dendritic cells and activates the JAK1/STAT3 cascade, in which STAT3 is phosphorylated by JAK1 and activates anti-inflammatory genes, reducing antigen presentation [69,70]. IL-10 regulates the regeneration of injured tissues and reduces the NPP caused by chronic restraint injuries to the sciatic nerve [71]. Systemic inflammation can be induced by the intraperitoneal injection of lipopolysaccharide (LPS) in WT mice and IL-10R1-deficient mice, and IL-10 has been found to bind to its receptor on sensory neurons to downregulate CCL2 for immunomodulation [70].

Chemokines are a superfamily of small proteins that signal through G-protein-coupled receptors, and their receptors are very important for the regulation of inflammation in the nervous system [72]. Neuroinflammation plays a key role in the pathogenesis of neuropathic pain, and inflammatory mediators such as CCL2 bind to receptors located in the PNS to cause pain [73]. CCL2 shows chemotactic activity for monocytes and basophils, and it regulates migration and infiltration of monocytes and macrophages. The CCL2 released from the synaptic vesicles of spinal neurons is a major mediator for local inflammation and pain after PNI. It promotes the recruitment of monocytes, neutrophils, T cells, and dendritic cells to the site of injury, exacerbating inflammation [74].

#### 2.3.2. Neurotrophic Factors

Neurotrophic factors are usually secreted by innervated target tissues (including neuroinflammatory cells), and they control the growth and survival of different neurons. During development, neurotrophic factors play a key role in nerve growth and neurodiversity in the PNS [75]. After PNI, target tissues and SCs secrete nerve growth factor (NGF) and brain-derived nerve growth factor (BDNF) to reduce neuronal death. NGF can bind to receptors to reduce neuronal apoptosis and promote the repair and regeneration of injured peripheral nerves [76]. BDNF in the brain is mainly synthesized by the cell bodies of neurons and glial cells. BDNF plays a crucial role in promoting nerve growth and maturation during developmental stages as well as regulating synaptic transmission and plasticity during adulthood. Su et al. used a composite nerve conduit containing sustained-release BDNF to bridge the sciatic nerve and verified that BDNF promotes the regeneration of the sciatic nerve in rats [77].

The roles of the above-described factors involved in inflammation in peripheral nervous system after injury are summarized in Table 2.

### 2.4. Inflammasomes

Inflammasomes are protein complexes of the innate immune system that initiate inflammation in response to exogenous pathogens or endogenous danger [78], and the most intensively studied is the NLRP3 inflammasome that is composed of NLRP3 protein, apoptosis-associated speck-like protein (ASC), and pro-caspase-1 [79]. The activation of the inflammasome is accompanied by the activation of caspase-1 that promotes the secretion of inflammatory cytokines by cleaving inactive pro-peptides, pro-IL-1β, and pro-IL-18 into mature cytokines while inducing inflammatory cell death, thereby promoting an inflammatory response [66,80]. Inflammasomes are involved in the pathogenesis of inflammation and NPP after nerve injury [81]. In order to investigate the relationship between NLRP3 inflammasome and nerve recovery, Cui et al. found that inflammasome-associated factors, such as IL-1β, IL-18, ASC, and caspase-1, were significantly up-regulated after sciatic nerve injury, and that, compared with WT mice, NLRP3-KO mice showed faster neurologic recovery after sciatic nerve injury [82]. Nerve injury is often accompanied by nerve pain, which is exacerbated by the hyperactivation of NLRP3 inflammasomes. In SCs, NLRP3 inflammasome plays a vital role in promoting regeneration after PNI, which is responsible for the demyelination of peripheral nerves [79]. Following PNI, macrophages are recruited to the site of injury. The activation of NLRP3 inflammasomes promotes the recruitment of macrophages and neutrophils by regulating the release of IL-33 [83]. And, NLRP3 inflammasomes in SCs can also influence the polarity of macrophages [79].

### 2.5. Complement System

The complement system is present in normal fresh human and vertebrate sera as non-specific globulins associated with enzyme activity. The complement system, also known as the serum-effective system, is part of the innate immune system, which is not only involved in the inflammatory response [84] but also promotes tissue regeneration and repair [85]. The complement system, which consists of more than 40 soluble and membrane-associated components, modulators, and receptors, also contributes to recovery from infection or injury, regulates adaptive immune responses, and limits microbial infections [86]. Pathogens can activate the complement system through three distinct pathways: the classical activation pathway, the lectin pathway, and the alternative pathway. However, the dysregulation, injury, unintentional activation or over-activation of complements may contribute to the pathogenesis of certain autoimmune neurological disorders and may even lead to neurodegenerative diseases such as Alzheimer’s disease [87,88].

After injury in the PNS, the complement cascade is quickly (within 1 h) activated locally at the site of damage. Myelin proteins can activate the complement system through the classical and the alternative pathway in an antibody-independent manner, and the myelin phagocytosis by macrophages is mediated by the complement via complement type 3 receptor (CR3). Furthermore, the membrane attack complex (MAC) is essential for rapid WD and the efficient clearance of myelin after acute nerve damage in the PNS [89,90]. Complement activation during WD may be a “double-edged sword” in the process of nerve regeneration. On the one hand, it may cause permanent damage to non-specific tissues directly through the MAC or indirectly through macrophages and their toxic mediators; on the other hand, complement mediation may favor the termination of inflammation and facilitate nerve recovery by disassembling damaged axons and myelin sheaths, removing debris, and delaying the secretion of anti-inflammatory cytokines from macrophages [89,90].

## 3. Relationship between Inflammation and Nerve Regeneration and Applications

Neuroinflammation is a self-protective mechanism of an organism, through which the nervous system protects itself from pathologic injury [8,91]. Inflammation can protect nerves and promote axonal regeneration [10]. Peripheral nerves have high regenerative capacities, but the severity of the PNI determines the success of nerve regeneration [92]. After nerve injury, distal axons and myelin sheaths are degenerated, neurotrophic factors are produced in vivo, corresponding receptors are increased, and neurons activate several different signaling pathways, contributing to functional growth cone regeneration. Chemokines produced by SCs influence axon growth, and macrophages and SCs remove damaged tissue debris from the wound. Lymphocytes are infiltrated after nerve injury and produce a variety of neurotrophic factors such as PDGF, insulin-like growth factor-1 (IGF-1), NGF, BDNF, and NT-3 [93]. In this way, the lymphocytes that reach the site of injury become a source of these growth factors. WD degrades the entire axonal structure and prepares good conditions for newborn axons [94]. For example, after nerve injury, the activation of macrophages to remove myelin debris is necessary for remyelination. The absence of specific inflammatory response products may also impede nerve recovery, as TNF-α deficient mice exhibit a significant worsening of nitric oxide (NO)-induced neuronal excitotoxicity [95]. Following spinal cord injury, neutrophils are rapidly mobilized and largely infiltrate the spinal cord, and neutrophil-deficient mice show worse neurological recovery using anti-Ly6G/Gr-1 antibodies [96].

In the early stages after nerve injury, inflammation is beneficial because it removes tissue debris and increases levels of neurotrophic factors. However, when the inflammatory response persists, the inflammatory cells release large amounts of inflammatory cytokines, causing further damage to the microenvironment, which is not conductive to nerve recovery and even damaging to healthy nerves. Therefore, excessive inflammation is detrimental to the recovery of body functions after nerve injury [97]. Although WD-derived inflammation is associated with several beneficial effects for axon elongation, the shutdown of this inflammatory process is also essential for nerve regeneration. Uncontrolled inflammation is the underlying reason for innumerable nerve pathologies, including neuropathic pain and autoimmune diseases. Drugs are usually used to relieve or suppress the inflammation due to a nerve injury. The vitamin B complex can be used to inhibit local inflammation after PNI and thereby promote the recovery of nerve function [98]. The vitamin B complex decreased the expression of proinflammatory macrophages and the number of M1 macrophages, and increased the expression of anti-inflammatory cytokines and the number of M2 macrophages, thus contributing to the resolution of neuroinflammation. Albay et al. [99] investigated the effect of vitamin E and vitamin B12 on functional recovery in a rat model of sciatic nerve crush injury and confirmed that vitamin E combined with vitamin B12 was effective in treating sciatic nerve injuries. Scaricamazza et al. [100] used Trimetazidine in the treatment of amyotrophic lateral sclerosis, a neurodegenerative disease, and found that the drug prevented the degeneration of the neuromuscular junction and reduced inflammation in the peripheral nerves.

With the rapid developments made over time, people are beginning to realize that active ingredients extracted from traditional Chinese medicine can be applied to nerve regeneration. Berberine, an isoquinoline alkaloid derived from natural herbs, was experimentally verified by Zhang et al. [101] to alleviate the local inflammation caused by lacerations and to promote nerve regeneration, while facilitating PI3K and Akt activation. Cordycepin, abundant in wild Cordyceps chrysosporium, has anti-inflammatory and neuroprotective effects. It contributes to the regeneration of myelin sheaths and inhibits the expression of pro-inflammatory factors [102]. Yang et al. [103] investigated the effects of Ramulus Cinnamomi extract on inflammation and its mechanism, and found that it could inhibit signaling pathways such as TLR4/MyD88, thereby relieving neuroinflammation. Dietary therapy has been found as a complementary means to treat inflammation. In a gastric ulcer model, honey decreased serum levels of IL-1β and TNF-α, and increased IL-10 levels [104]. Qinghui Gu found that in an in vitro simulated model of inflammation, the polysaccharides BFP60 and BFP80 extracted from Bletilla formosana significantly reduced the production of inflammatory cytokines such as TNF-α and mediator NO in LPS-induced RAW6.1 cells [105].

With the development of science and technology, some novel materials or fusions of materials with different properties have been discovered to capitalize on their benefits and inhibit inflammation, thereby promoting nerve regeneration. Prussian blue, approved by the U.S. Food and Drug Administration (FDA), is widely used as an antidote in clinics for thallium poisoning [106]. Prussian blue has multi-enzyme activities, including peroxidase, catalase, and superoxide dismutase activities, that can effectively inhibit, mitigate, or even scavenge reactive oxygen species [106]. It can also attenuate oxidative stress, inhibit apoptosis in vitro and in vivo, and resist inflammation [107]. Prussian blue nanoparticles are often used as drug carriers due to their good biocompatibility and high efficiency in drug loading [108]. It has been shown that Prussian blue nanoparticles carrying mesenchymal stem cells enable the cells to survive under high oxidative stress, and also enhance paracrine effects such as cytokines and immunomodulation [109]. In a study by Yujuan Tian, mesoporous Prussian blue nanoparticles carrying baicalein were used to treat bacterial-induced periodontitis, and these nanoparticles promoted macrophage conversion to an M2 type, decreased IL-1β and TNF-α, and scavenged ROS, thereby reducing inflammation [110].

## 4. Conclusions and Perspectives

After PNI, a variety of cells in the body interact with cytokines, chemokines, and trophic factors. As research has progressed, it has become increasingly clear that the repair and regeneration after nerve injury are linked to inflammation. In the early stages of injury, the body’s innate immune system relies on its own protection. Immune cells and various inflammatory mediators secreted contribute to the removal of necrotic tissue debris and promote nerve regeneration. Conversely, excessive inflammation in the later stages is detrimental to the recovery of nerve function, and in severe cases, even leads to neurological diseases. Therefore, the physiological role of inflammation after nerve injury should be fully understood to avoid the unfavorable factors of inflammation, and the positive effects of inflammation should be used to promote repair and regeneration after PNI.

## Figures and Tables

**Table 1:** The roles of cells in inflammation in peripheral nervous system after injury.

Cells	Functions	References
Macrophages	maintain homeostasis, repair wounds, tissue regeneration, phagocytose fragments, secrete factors and pro-inflammatory pain mediators, involve in axonal regeneration, promote neuroglial cells migration and proliferation, and produce VEGF-A	[6,26,28,29,34,35]
Neutrophils	phagocytose debris, release cytokines, chemokines, and NP-1 to participate in immune response responses	[37,41,42]
Lymphocytes	Produce pro-inflammatory or anti-inflammatory cytokines and participate in the immune response after injury	[46,47]
Schwann cells	constitute the blood-nerve interface, regulate nerve injury, clear myelin sheath and recruit immune cells, downregulate myelination genes, activate a series of repair axon genes, process and present antigens	[48,51,54,55,56]

VEGF-A, vascular endothelial growth factor-A; NP-1, neutrophil peptide-1.

**Table 2:** The roles of factors involved in inflammation in peripheral nervous system.

Factors		Sources	Effect	Mechanism	References
Cytokines	IL-1β	Lymphocytes and macrophages	Pro-inflammatory	directly involved in the inflammatory response, activating NF-κB and promoting the expression of inflammatory factors	[57,58,59,63]
	TNF-α	Lymphocytes and macrophages	Pro-inflammatory	promote the secretion of pro-inflammatory mediators activate the immune system	[59,67]
	IL-10	Macrophages and B cell	Anti-inflammatory	regulate the regeneration of damaged tissues, inhibit the secretion of pro-inflammatory factors and the expression of MHC, and inhibit the activity of immune cells	[59,69,70,71]
Chemokines	CCL2	Lymphocytes and macrophages	Pro-inflammatory effect	cause neuropathic pain, recruit cells, exacerbate inflammation	[73,74]
Neurotrophic factors	NGF	Target tissue and Schwann cells	Promoting growth	reduce neuronal apoptosis and promote nerve repair and regeneration	[76]
	BDNF	Target tissue and Schwann cells	Promoting growth	promote neural growth during development, regulate synaptic transmission and plasticity in adulthood	[77]

IL-1β, interleukin-1β; TNF-α, tumor necrosis factor-α; IL-10, interleukin-10; CCL2, C-C motif chemokine ligand 2; NGF, nerve growth factor; BDNF, brain-derived nerve growth factor; NF-κB, nuclear factor-κB; MHC, major histocompatibility complex.
